# Malignant phyllodes tumor of the breast with predominant osteosarcoma and chondrosarcomatous differentiation: a rare case report and review of literature

**DOI:** 10.3389/fonc.2024.1372710

**Published:** 2024-04-19

**Authors:** Wenfang Li, Qin Ou, Yingdong Li, Linlin Yuan Yuan

**Affiliations:** ^1^ Taihe Hospital, Hubei University of Medicine, Shiyan, China; ^2^ Department of Pathology, School of Basic Medical Sciences, Hubei University of Medicine, Shiyan, Hebei, China; ^3^ Department of General Surgery, Taihe Hospital, Hubei University of Medicine, Shiyan, Hubei, China

**Keywords:** malignant phyllodes tumors, breast tumor, osteosarcoma, chondrosarcoma, thoracic oncology

## Abstract

**Background:**

Phyllodes tumors (PTs), which account for less than 1% of mammary gland tumors, composed of both epithelial and stromal components. If a malignant heterologous component is encountered, PT is considered malignant. Malignant phyllodes tumors (MPTs) only account for 8% to 20% of PTs. We report a case of MPT with osteosarcoma and chondrosarcoma differentiation and review the literature to discuss the differential diagnosis and therapy.

**Case presentation:**

A 59-year-old Chinese woman come to our hospital because of a palpable mass she had had for 1 months in the left breast. Preoperative core needle biopsy (CNB) was performed on the left breast mass on January 11, 2023. Pathological diagnosis was malignant tumor, the specific type was not clear. Mastectomy and sentinel lymph node biopsy of the left breast was performed. No metastasis was found in 3 sentinel lymph nodes identified by carbon nanoparticles and methylene blue double staining. Heterologous osteosarcoma and chondrosarcomatous differentiation of phyllodes tumor were observed. Immunohistochemistry: spindle tumor cells ER(-), PR(-), HER-2(-), CK-pan(-), CK7(-), CK8(-), SOX10(-), S100(-), and MDM2(-), CK5/6(-), P63(-), P40(-) were all negative. CD34:(+), SATB2(+), P53(90% strong), CD68 (+), Ki-67(LI: about 60%). No ductal carcinoma *in situ* was found in the breast. Fluorescence *in situ* hybridization (FISH) indicated USP6 was negatively expressed on formalin-fixed, paraffin-embedded (FFPE) tissue sections.

**Conclusion:**

MPTs are rare, and heterologous differentiation in MPTs is exceedingly rare. It could be diagnosed by pathology when metaplastic carcinoma, primary osteosarcoma, or myositis ossificans were excluded. This case could help clinicians to improve the prognosis and treatment of this disease.

## 1 Introduction

Phyllodes tumors (PTs), which account for less than 1% of mammary gland tumors, composed of both epithelial and stromal components (1). Malignant phyllodes tumors (MPTs) only account for 8% to 20% of PTs (2). The World Health Organization has subcategorized PTs into benign, borderline, and malignant categories on the basis of 5 histological parameters: stromal cellularity, stromal atypia, tumor margins, mitotic activity, and stromal overgrowth (3). MPTs are characterized by marked stromal cellularity, stromal growth, nuclear atypia, increased mitotic activity (≥10 per 10 high power fields), and infiltrative tumor margins (4). Moreover, the presence of heterologous sarcomatous elements such as osteosarcoma, chondrosarcoma, or liposarcoma within the tumor were frequently observed (5). Due to the limitation of rare incidence, it is difficult to proceed randomized trials and prospective cohort studies for MPTs with heterologous sarcomatous elements (6).

This study describes a case/patient with osteosarcomatous and chondrosarcomatous heterologous elements within a MPT based on detailed imaging and histopathologic records, and we review the literature to describe the characteristics and therapy of MPT with osteosarcoma and chondrosarcomatous differentiation.

## 2 Clinical data

**Figure 1:**
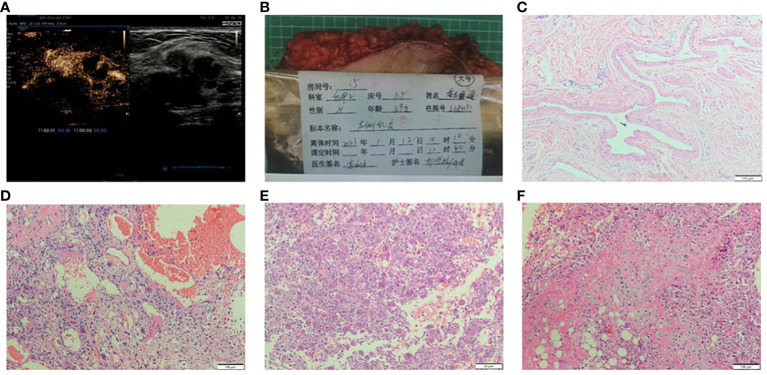
**(A)** Breast US images revealed a hypoechoic irregular mass in the breast left upper quadrant, with a size of about 23×25×19mm, the boundary was not uneven and no strong punctate echo was observed. The combination of contrax-enhanced ultrasound suggested the mass uneven enhancement in the arterial phase, unsmooth boundary, slow regression in the venous phase, and obvious enlargement in the lesion area. **(B)** The whole excised tissue of the left breast. **(C)** The resected specimen showed leaf-like (phyllodes) epithelial pattern (HE×100). **(D)** Stromal overgrowth, tumor giant cells and many abnormal mitosis are noted (HE×100). **(E)** The stroma showed a tumor osteoid rimmed by tumor cells along with osteoclastic giant cells, osteosarcoma differentiation (HE×100). **(F)** The section showed tumor cells with chondrosarcomatous differentiation, the chondrocyte density is different, the cell atypia is obvious, the nuclear mitosis and cartilage islands can be observed (HE×100).

Patient, ×××, female, 59 years old, due to “presented with a mass in the left breast for more than 1 month”. On January 7, 2023, she was admitted to the breast surgery Department of Taihe Hospital, Hubei University of Medicine. In the past 1 month, she felt a mass in the left breast, and the nipple was not bleeding or leaking. The patient had a history of hypertension for 30 years and cerebral hemorrhage for more than 1 year. In 2013, she underwent “left breast mass resection” in our hospital, and the pathological report was cystic hyperplasia (left breast). Clinical physical examination: a hard mass of about 3.0×2.0cm in size could be detected at 4cm from the nipple in the upper outer quadrant of the left breast, with no obvious tenderness, non-smooth surface, unclear boundary, and limited motion. No obvious abnormalities in the contralateral breast were found. There is no obvious mass in the bilateral axilla. The color ultrasound of the breast showed that there was a low-echo mass located at the edge of the mammary gland at 2 points in the left upper quadrant, with a size of 23×25×19mm, the boundary was not clear, and the shape was irregular, and no strong punctate echo was observed (**Figure 1A**). The combination of contrax-enhanced ultrasound suggested hypoechoic mass in the left breast, uneven enhancement in the arterial phase, unsmooth boundary, slow regression in the venous phase, and obvious enlargement in the lesion area identified as BI-RADS Category V. Reactive hyperplasia of bilateral axillary lymph nodes.

Preoperative core needle biopsy (CNB) was performed on the left breast mass on January 11, 2023. Pathological diagnosis was malignant tumor, the specific type was not clear, and further diagnosis was to be made after surgery. The patient given up breast conserve surgery. Mastectomy and sentinel lymph node biopsy of the left breast was performed. No metastasis was found in 3 sentinel lymph nodes identified by carbon nanoparticles and methylene blue double staining. Pathological examination results: the size of the left breast was 23×18×3.0cm, and the fusiform skin was attached, the size of which was 19.5×7.0cm (**Figure 1B**). The size of the tumor was 3.0cm×2.5cm×2.2cm. The section was grayish-white and slightly hard in nature, and the boundary was poorly defined or poorly circumscribed. The tumor was 2.2cm away from the skin and 0.5cm away from the deep margin. TNM stage given was pT2N0M0.

**Figure 2:**
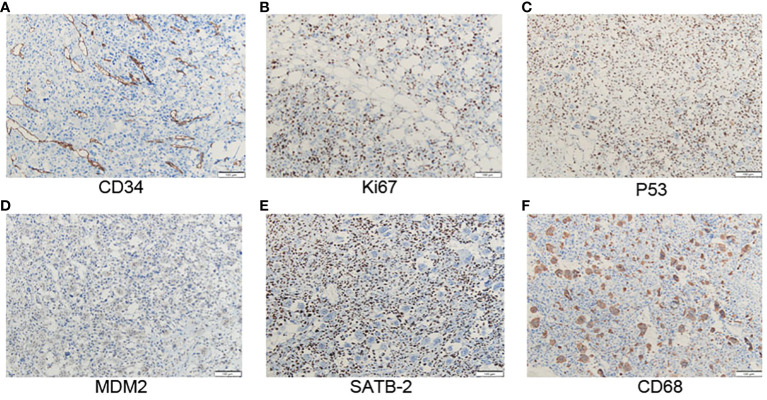
**(A)** Positive staining with CD34 in atypical stromal cells (x100). **(B)** The positive staining with Ki-67 in atypical stromal cells is about 60% (x100). **(C)** The positive staining with p53 in atypical stromal cells is about 90% (x100). **(D)** MDM2 is negative expressed in stromal cells (x100). **(E)** SATB2 is positively stained in atypical stromal cells (x100). **(F)** CD68 is expressed in osteoclastic giant cells (x100).

The tumor is composed of two components: (1) The normal breast lobular structure is disordered or disappeared. The tumor has a lobulated mass (**Figure 1C**), which is composed of epithelial and stromal components. The epithelial cells are columnar or cuboidal without obviously atypia. Stromal cells are fusiform, the cytological atypia was obvious (**Figure 1D**), with 10 mitotic images/10HPF. And the epithelioid stromal cells are interspersed with short fusiform plump cells and mononuclear/multinucleoma giant cells. Local stromal cells form pseudoadenoid or clumps or nests. Mesenchymal myxoid changes in some areas of the tumor, the mesenchymal cells are sparse, epithelioid or spindle, and scattered tumor giant cells are also seen. About 1/5 of the tumor interstitial tissues showed fibrosis or hyalinoid degeneration. (2) Heterogenic components of tumor mesenchyma were observed: cartilage and bone tissue. The cartilage tissue presented cartilaginous islands of different sizes. The stroma showed a tumor osteoid rimmed by tumor cells along with osteoclastic giant cells, osteosarcoma differentiation. Tumor cells surrounded trabeculae, and the cell atypia was significant, showing mitotic images (**Figure 1E**). Chondrocytes of different density were observed with obvious cell atypia, and mitotic images (**Figure 1F**). ③ Immunohistochemistry: Spindle tumor cells ER(-), PR(-), HER-2(-), CK-pan(-), CK7(-), CK8(-), SOX10(-), S100(-), and MDM2(-), CK5/6(-), P63(-), P40(-) were all negative. CD34:(+), SATB2(+), P53(90% strong), CD68 (+), Ki-67(LI: about 60%) (**Figure 2**). ④ No ductal carcinoma *in situ* was found in the breast, and no metastasis were found in axillary lymph nodes. Pathological consultation advice of Union Hospital Affiliated to Tongji Medical College of Huazhong University of Science and Technology: Malignant (breast) phyllodes tumor with osteosarcoma and chondrosarcoma differentiation was considered.

### 2.1 Fluorescence *in situ* hybridization

Two-color separation probe kit was purchased from Lbp Medicine Science & Technology (Guangzhou, China) which was adopted to detect USP6 on formalin-fixed, paraffin-embedded (FFPE) tissue sections. The probe is located on chromosome 17p13.2, the proximal (proximal centromere) probe marks red fluorescence, and the distal (proximal telomere) probe marks green fluorescence. Fluorescence *in situ* hybridization (FISH) testing procedure was in strict accordance with the standardized steps: The 3-5μm thick tissue was sliced, incubated for 2 h at (65 ± 5)°C, dewaxed and hydrated, deionized boiled at (100 ± 5)°C for 20 min, digested by pepsin for 15 min, dripped with a probe, denatured at 85°C for 5 min, and hybridized at 37°C for 10-18 h. Nuclear restaining of 4 ‘, 6⁃diamidino⁃2 ‘phenylindole (DAPI) under fluorescence microscope.

**Figure 3:**
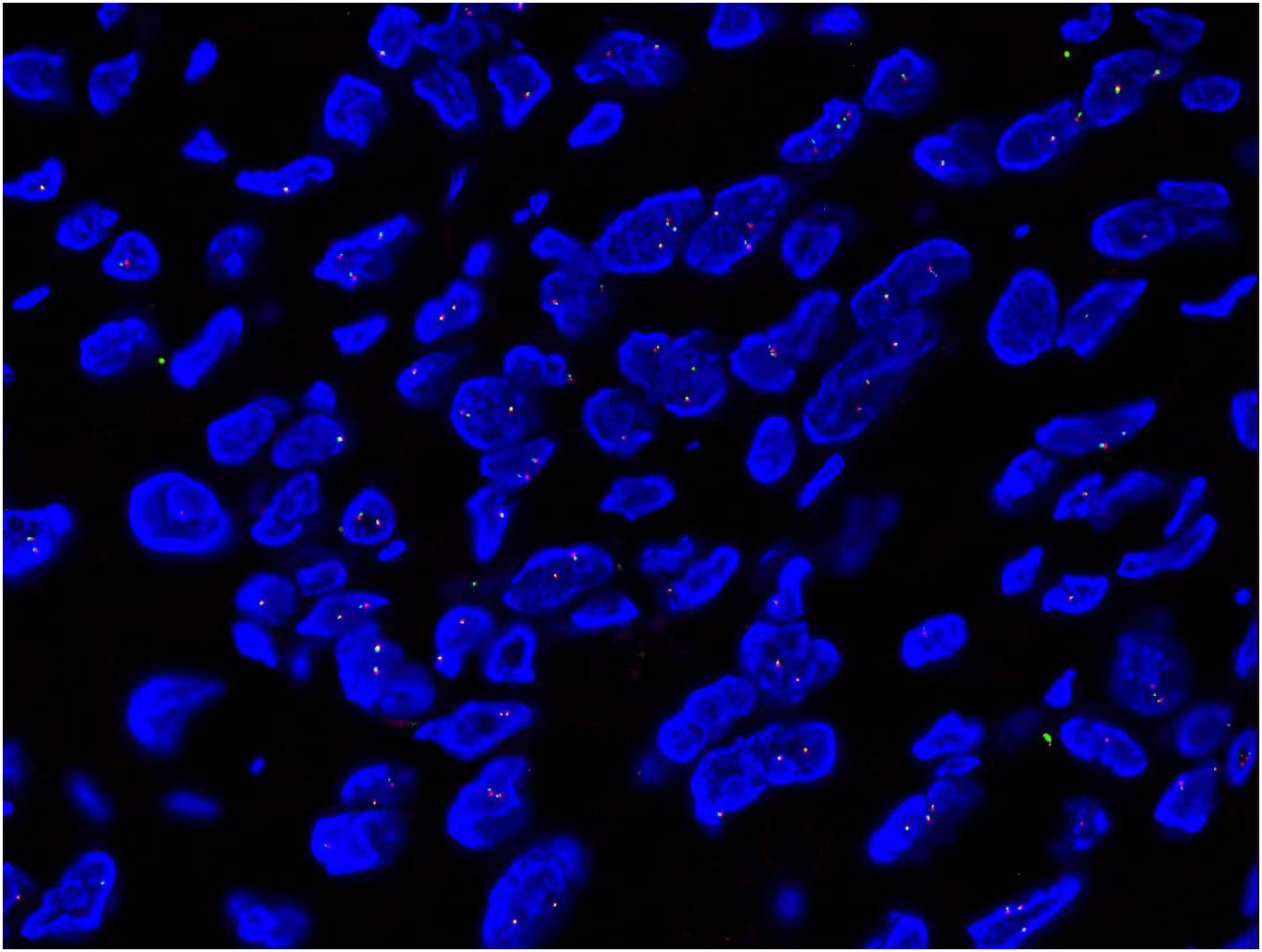
FISH showed USP6 positively rate was 2% which indicated negative expression on sections (x1000).

Results Interpretation: The FISH results were interpreted independently by two experienced pathologists: 200 neoplastic cells in a blind fashion using an Olympus BX53 fluorescence microscope (Japan) were counted, the distance between red signal and green signal was > 2 signal points as positive cells, and the proportion of positive cells was > 10% as positive for separation and rearrangement (7). FISH showed USP6 positively rate was 2% which indicated negative expression on sections (**Figure 3**).

After radical resection of the left breast, anthracycline and ifosfamide chemotharapy were adopted for four cycles, and the patient still survived without any recurrence after eight months of follow up.

## 3 Discussion

MPTs are identified when the tumor exhibits marked stromal nuclear pleomorphism, stromal overgrowth, with infiltrating borders, markedly increased stromal cellularity, stromal overgrowth with severe nuclear atypia (8). Higher malignancy grade, presence of heterologous elements, younger age, larger tumor size, and recent rapid tumor growth are poor prognostic factors for MPTs of the breast (9). Heterologous differentiation in MPTs is exceedingly rare, but there are MPTs with chondrosacomatous or osteosarcomatous differentiation reported (10). MPTs with the histological osteosarcomatous subtype increase mortality by 33% (11). MPTs accompanied by osteosarcoma only accounts for 1.3% of phyllodes tumors in the breast (12). According to Silver et al, MPTs with osteosarcomatous components are potentially more aggressive and could spread to the lung, bone, brain, contralateral breast. In our patient, we describe a MPT with osteosarcoma and chondrosarcomatous differentiation.

Stromal and epithelial cells of the breast tissues are the mainly components to develop malignancy. According to the different malignancy of both cells, it could been diagnosed as benign or malignant diseases (13). In the present case, most of the stromal cells were fibrosarcoma-like interlacing fascicles of spindle cells with stromal overgrowth. Multinucleated stromal giant cells have been rerorted in phyllodestumors. Focal areas of osteosarcoma and/or chondrosarcomatous differentiation were hemothera. MPT is also diagnosed when malignant heter-elements such as osteosarcoma, chondrosarcoma, and rhabdomyo-sarcoma are present even if the other features are absent. Chondrosarcomatous component even constituted over 80% of the tumor volume which is indeed rare (14).

In breast tumor, it is found that osteosarcoma or chondrosarcoma might occur in 3 different diseases: primary osteosarcoma of the breast as with a pure osteosarcoma or chondrosarcoma, as the stromal component of a histologically MPT, or as osteosarcomatous or chondrosarcomatous differentiation in a metaplastic carcinoma (15). Primary osteosarcoma of the breast is also a rare primary breast tumor which accounts for only 1% of breast tumors and < 5% of all osteosarcomas (16). The possibility of metastasis of osteosarcoma from other sites should be ruled out first, and it has the following two characteristics in histology: neoplastic osteogenesis or osteoid matrix; No epithelial component. The immunophenotype of primary osteosarcoma was strongly positive for vimentin, strongly positive for CD68 in osteoclastic multinucleated giant cells, and negative for ER, PR, Her-2 and epithelial markers (17). Some evidence suggests that MPTs with osteosarcomatous hemotherapy on are more aggressive, but compared with primary osteosarcomas in general, they have a much lower risk of metastasis (18).

Metaplastic carcinoma of the breast also have sarcoma like elements, including spindle cell sarcoma, chondrosarcoma, osteosarcoma, rhab-domyosarcoma, or a mixture of them (19). However, high molecular weight cytokeratin and p63 usually were positive in metaplastic carcinoma. These could be helpful in hemotherapyio these two tumors (20). It is also reported that p63 could also been diffusely and focally expressed in MPT (21). In this patient, we could also observe sporadic p63 expression, but lack of CK expression, these molecular markers help to eliminate metaplastic carcinoma or primary osteosarcoma. Special AT-rich sequence-binding protein 2 (SATB2) induces local chromatin loops to facilitate transcription. SATB2 immunostaining is commonly used as a marker for colorectal adenocarcinoma and osteosarcoma (22). In our patient, SATB2 expression was strong positive, which indicated osteosarcoma tissues. CD34 is also observed in MPT (23), this is in line with our case, that CD34 is positively expressed.

Another differential diagnosis of MPT is breast myositis ossificans. Myositis ossificans is defined as a self-limiting pseudotumor composed of reactive hypercellular fibrous tissue and bone. USP6 rearrangements have been identified as a consistent genetic driving event in aneurysmal bone cyst and nodular fasciitis (24). It is therefore an integral part of the diagnostic workup when dealing with (myo)fibroblastic lesions of soft tissue and bone. USP6 rearrangement provided evidence of a relationship with nodular fasciitis and aneurysmal bone cyst (25). In our patient, the USP-6 is negative and supplies another strong proof to eliminate the diagnosis of myositis ossificans.

Recent next generation sequencing analyses had revealed novel genetic alterations in PT but lacked a further hemotherapyion of their relationship to different PT features and outcome (26). Malignant progression is associated with heterocytogenetic abnormalities, including MYC amplification, p53 mutation, increases in chromosomes 1q, 5p, 7 and 8, and loss of 6q, 9p, 10p, 13q, 16q and 19 (27). MED12 mutations are associated with alterations in related genes in the Wnt, TGFB, and THRA pathways (28, 29). Lin et al. demonstrated that ALDH1 and/or GD2 markers could be used for cancer stem cell research in patients with MPT (30).

The epithelial-mesenchymal transition (EMT) increased with the progression of MPTs tumor grade. Nuclear expression of the EMT proteins TWIST and Foxc2 is associated with increased tumor grade and deterioration of histological features (31, 32). Additional mutations or copy number alterations in known cancer driver genes NF1, RB1, TP53, PIK3CA, ERBB4, and EGFR have been identified in borderline and malignant MPTs through next-generation sequencing (33, 34). These molecules provide an important biological basis for the occurrence and development of MPTs, and provide a theoretical basis for molecular diagnosis and therapeutic targets in clinical practice.

Metastatectomy has been correlated with increased overall survival (of 25.9 versus 9.9 months; *P* = .01) in MPT (35). The definitive treatment for phyllodes tumor is wide surgical excision with at least 1-2 cm of negative margins, or mastectomy, depending on the size of the tumor and the patient’s breast size (36). Histological size ≥45 mm and dense stromal cellularity were demonstrated as histological risk factors of local recurrence of PT (37). Radiotherapy has often been associated with palliation and pain control in metastatic, malignant neoplasia. Anthracycline containing chemotherapy regimens has been associated with improved overall survival (22.4 months versus 13.2 months; *P* = .040). Anthracycline and alkylating agent-based combination regimens were most frequently administered (38). In the present case, metastatectomy was performed, anthracycline and ifosfamide hemotherapy were adopted, and the patient still survived without any recurrence.

Our case report illustrates that breast osteosarcoma and chondrosarcoma differentiation originating from an MPT is remarkably difficult to diagnose and manage. The standard treatment comprises complete excision of the tumor with wide margins or total mastectomy. A multiple oncology gene mutations happen and promote the malignant progression. The adjuvant therapy is still controversial due to the lack of multi-center large patients records and suitable clinical trials. Further research must be conducted to elucidate accurate diagnosis and clarify the best treatment for these tumors.

## Data availability statement

The original contributions presented in the study are included in the article/Supplementary Material. Further inquiries can be directed to the corresponding author.

## Ethics statement

The studies involving humans were approved by Ethics Committee of Shiyan Taihe Hospital (NO.2023KS55). The studies were conducted in accordance with the local legislation and institutional requirements. Written informed consent for participation in this study was provided by the participants’ legal guardians/next of kin. Written informed consent was obtained from the individual(s) for the publication of any potentially identifiable images or data included in this article.

## Author contributions

WL: Writing – review & editing, Writing – original draft, Resources, Funding acquisition. QO: Writing – original draft, Project administration, Methodology, Investigation, Data curation. YL: Writing – original draft, Methodology, Data curation. LY: Writing – review & editing.
